# Iron starvation confers azole tolerance in *Aspergillus fumigatus* hyphae via mitochondrial function modulation

**DOI:** 10.1128/mbio.00896-26

**Published:** 2026-06-15

**Authors:** Longyun Cong, Zhengyu Lin, Qiuchen Li, Wei Wang, Shizhu Zhang

**Affiliations:** 1 Jiangsu Key Laboratory for Pathogens and Ecosystems, College of Life Sciences, Nanjing Normal University 12534, Nanjing, Jiangsu, China; Universidade de Sao Paulo Campus de Ribeirao Preto, Ribeirao Preto, Sao Paulo, Brazil

**Keywords:** *Aspergillus fumigatus*, iron starvation, azole tolerance, mitochondrial function

## Abstract

Antifungal tolerance, a transient survival state distinct from genetic resistance, poses a significant challenge to antifungal therapy. However, the factors that induce antifungal tolerance and their underlying mechanisms remain poorly understood. Here, we demonstrate that iron starvation exerts a paradoxical effect on azole susceptibility in the human pathogenic filamentous fungus *Aspergillus fumigatus* by specifically increasing azole tolerance in hyphae while reducing the minimal inhibitory concentration against both conidia and hyphae. This hyphal tolerance could be quantified using a newly developed protoplast-based time-kill assay. The minimum duration required to kill 99% of hyphal cells (MDK_99_) following azole treatment was significantly prolonged under iron-starved conditions. Further investigations reveal that iron starvation impairs mitochondrial function by reducing the activity of mitochondrial electron transport chain (ETC) complexes I and III. This attenuation leads to a marked reduction in two key hallmarks of azole-induced hyphal death, including reactive oxygen species accumulation and carbohydrate patch formation. Supporting this mechanism, genetic deletion of *mba1*, a critical mitochondrial assembly factor essential for the proper biogenesis of ETC complexes I and III, confers azole tolerance in *A. fumigatus* hyphae even under iron-replete conditions. Importantly, this iron starvation-mediated azole tolerance could be restored through supplementation with the mitochondrial cofactor coenzyme Q10. Overall, these findings identify iron availability as a key environmental modulator of azole fungicidal effect and reveal potential therapeutic targets to counteract this antifungal tolerance in invasive fungal infections.

**IMPORTANCE:**

*Aspergillus fumigatus* undergoes an obligate life cycle with distinct morphotypes, and hyphae represent the predominant morphological form of the fungus during invasive pulmonary aspergillosis. However, owing to the multinucleate nature and pronounced physiological heterogeneity of hyphae, it is challenging to achieve quantitative and effective assessment of their drug susceptibility. In this study, by developing a protoplast release-based time-kill assay, we uncovered that iron starvation confers azole tolerance in *A. fumigatus* hyphae. Importantly, mechanistic investigations further revealed that supplementation with the mitochondrial cofactor coenzyme Q10 restores fungicidal activity of azoles against *A. fumigatus* hyphae under iron-starved conditions. Overall, this study underpins the elucidation of environmental factor-antifungal drug tolerance associations and offers targeted strategies against this tolerance.

## INTRODUCTION

*Aspergillus fumigatus* is a saprophytic filamentous fungus found in soils and compost. This mold is the primary cause of invasive aspergillosis, a severe and life-threatening infection with an estimated global incidence of 200,000 human cases per year and a mortality rate of 30%–95% (1–3). Azoles are recommended as the first-line medicine for the primary treatment of aspergillosis because of their high effectiveness against *A. fumigatus* and fewer side effects (2, 4). In most fungi, ergosterol is the most abundant sterol in the membranes. Azoles inhibit the lanosterol 14α-demethylase (CYP51/Erg11), which is one of the key enzymes in the ergosterol biosynthesis pathway (5, 6). In *A. fumigatus*, azoles first manifest as a rapid and potent fungistatic effect. Within approximately 1 h following azole exposure, hyphal growth is significantly suppressed, but cells remain viable (7). Azoles exhibit at least two killing modes of action against *A. fumigatus*. First, one of the mechanisms of the fungicidal activity to *A. fumigatus* is the result of a compensatory cell wall stress pathway following ergosterol depletion that results in carbohydrate patch formation with subsequent penetration of the plasma membrane and fungal killing (7). Second, like other antifungal drugs, azoles trigger the mitochondria-derived reactive oxygen species (ROS) production in *A. fumigatus* (8, 9). The induction of an oxidative burst may cause a toxic effect in fungi. Indeed, azoles have fungicidal activity on plant pathogenic fungi *Zymoseptoria tritici* and *Magnaporthe oryzae* by a combination of ROS-induced apoptosis and macroautophagy (10). Although azoles have multiple fungistatic and fungicidal mechanisms, prolonged treatment and environmental exposure to these drugs have led to the development of drug resistance over the past decades (11, 12). However, infection treatment failures still occur with clinically determined, drug-susceptible isolates (13–15). The findings indicate that complex interactions between the drug and the fungus, aside from drug resistance, may affect therapeutic responses as well.

Recently, the investigations on *Candida* isolates and *Cryptococcus neoformans* suggested that tolerance is a contributing factor to the discrepancy between minimal inhibitory concentration (MIC) data and clinical outcomes (16, 17). Antimicrobial tolerance describes the ability of microorganisms to survive the action of antimicrobial drugs that exceeds the MIC for extended periods (18, 19). In contrast to drug resistance, which is the reduced susceptibility of cells to a drug due to heritable genetic mutations, tolerance cannot be genetically inherited and reflects the ability of genetically sensitive cells to survive high doses of antimicrobial drugs (20–22). Thus, drug-tolerant cells do not show a change in MIC. Instead, this tolerance can be quantified using time-kill curve-based approaches (for example, minimum duration for killing 99% of cells [MDK_99_] assessment) as documented for several bacterial and fungal pathogens (23–26). Antibiotic tolerance has been extensively studied in bacteria; the physiological changes underlying drug tolerance are driven by the environment, including the host and microbial communities, and the bacterial genetic background (27, 28). Antifungal tolerance has been increasingly recognized in pathogenic yeasts (29–32). Notably, recent studies have revealed a link between azole tolerance and mitochondrial function in *A. fumigatus*, with complex III dysfunction being associated with elevated azole tolerance and reduced formation of carbohydrate patches (7, 33). However, in comparison to the studies in bacteria, environmental factors that can induce tolerance and the underlying mechanisms remain poorly understood in fungi, particularly for filamentous fungi, which present additional complexities when compared to single-celled microorganisms (19).

*A. fumigatus* undergoes an obligate life cycle comprising distinct morphotypes, each exhibiting unique characteristics. It reproduces through the production of airborne spores (conidia), a process termed conidiation (34). In immunocompromised individuals, inhaled *A. fumigatus* conidia can germinate and form filaments that penetrate and damage lung tissues. Notably, hyphae are the predominant fungal morphology observed during invasive pulmonary aspergillosis, while conidia are rarely detected (35). Thus, hyphal morphology better reflects the true physiological state during *in vivo* infection. However, research on *A. fumigatus* antifungal responses has historically been biased toward studies using conidia or early germlings, primarily due to their homogeneity and ease of handling in standardized assays (19, 36). In contrast, *A. fumigatus* hyphae are multinucleate and exhibit marked physiological heterogeneity (37), which leads to differential metabolic activity and drug sensitivity across distinct hyphal regions (38–40). Due to the complexity of the hyphal network, effective research methods for assessing hyphal drug sensitivity have been lacking, which has become a key knowledge gap in investigating the survival strategies of filamentous fungi under antifungal stress.

Iron is an essential trace element for microbial growth and metabolism; it is involved in many key physiological processes, such as respiratory chain electron transport, DNA synthesis, and redox reactions (41–43). In the host infection environment, iron ions are usually in a scarce state, as iron is tightly sequestered by proteins such as transferrin and lactoferrin (44, 45). Iron ions are typically present at concentrations below 1 µM in the extracellular microenvironment of the host lung (46), and are even lower in specific immune microenvironments, such as macrophage phagosomes (47). Previous studies have shown that modulating iron availability through chelators can alter the efficacy of antifungal drugs, producing effects ranging from synergistic enhancement to paradoxical antagonism, depending on the specific pathogen, chelator, and drug combinations (48, 49). This indicates a significant association between iron levels and antifungal drug therapy.

In this study, the effect of iron on the azole tolerance in *A. fumigatus* hyphae was investigated. To this end, we developed a protoplast release-based time-kill assay to quantify the hyphal MDK_99_. We demonstrate that iron starvation significantly enhances azole tolerance in *A. fumigatus* hyphae rather than in conidia. Mechanistically, iron starvation leads to the dysfunction of mitochondrial function, which in turn leads to a marked attenuation of two key hyphal death hallmarks of azole-induced hyphal death, namely ROS accumulation and cell wall carbohydrate patch formation. Importantly, this azole tolerance induced by iron starvation can be reversed by the supplementation of coenzyme Q10 (CoQ10). In conclusion, this study establishes a robust method for investigating hyphal tolerance in filamentous fungi and provides novel insights into the mechanistic understanding of antifungal tolerance.

## RESULTS

### Iron starvation reduces the metabolic inhibitory effect of azoles against *A. fumigatus* hyphae

**Fig 1:**
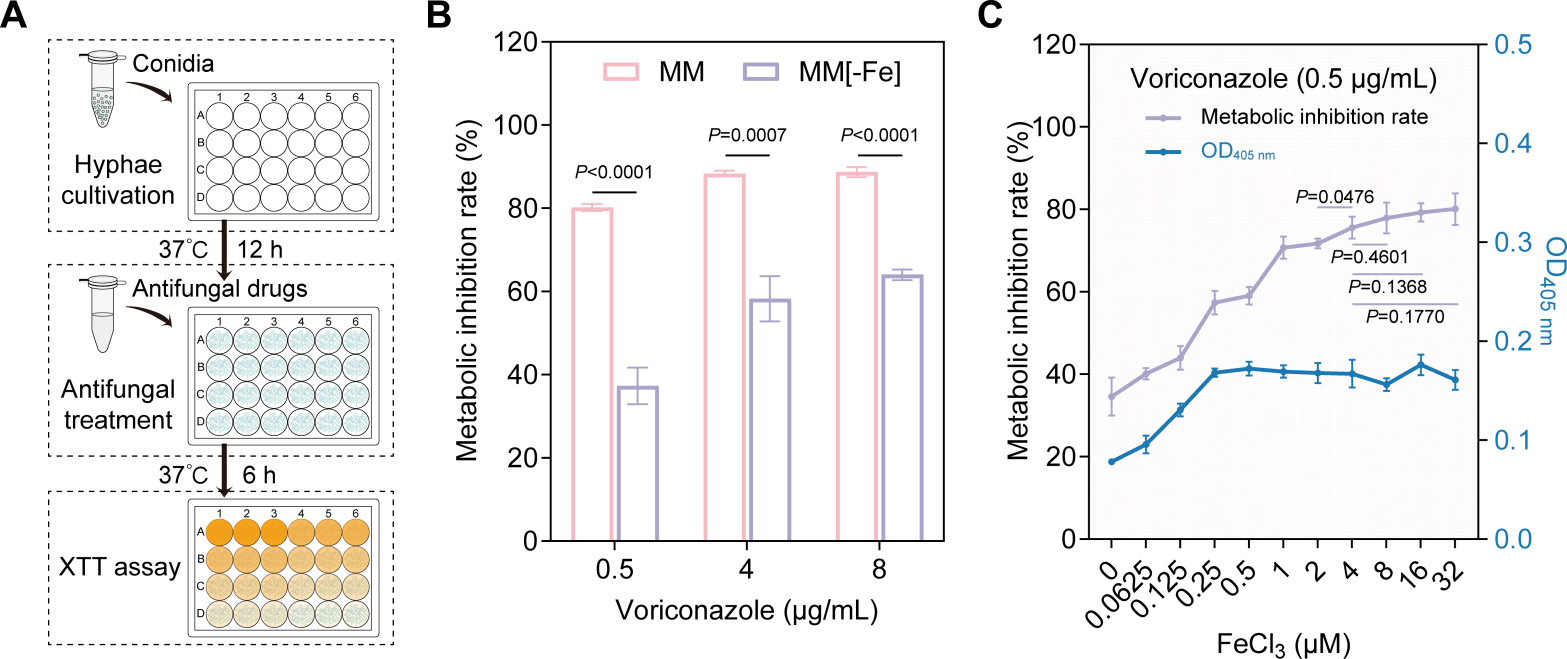
Iron starvation reduces the metabolic inhibitory effect of azoles against *A. fumigatus* hyphae. (**A**) Schematic diagram of the metabolic inhibition assay for azoles against *A. fumigatus* hyphae. (**B**) The metabolic inhibition assay of *A. fumigatus* hyphae treated with voriconazole for 6 h under iron-replete conditions (minimal medium, MM) and iron-starved conditions (MM without addition of iron, MM[-Fe]). (**C**) The metabolic inhibition rate and growth of *A. fumigatus* hyphae treated with voriconazole for 6 h under different iron concentrations. Conidia of the indicated strains were statically grown at 37°C for 12 h in the indicated medium and then treated with voriconazole at the indicated concentrations at 37°C for an additional 6 h. The metabolic activity was determined by XTT assay, and the metabolic inhibition rate was calculated as the relative metabolic activity of drug-treated cells compared to untreated cells under their respective conditions. Conidia of the indicated strains were statically grown at 37°C for 12 h in the indicated medium, and the growth was determined by assessing optical density at 405 nm. Experiments were conducted a minimum of three times, with each bar indicating the mean ± standard deviation (SD). Statistical analysis was performed using one-tailed, unpaired *t*-tests. *P* < 0.05 represents a significant difference, while *P* > 0.05 represents no significant difference.

To test the effect of antifungal drugs against *A. fumigatus* hyphae, an XTT (2, 3-bis-[2-methoxy-4-nitro-5-sulfophenyl]−2H-tetrazolium-5-carboxanilide)-based metabolic activity quantification was used. *A. fumigatus* conidia were first cultured in the indicated medium for 12 h to form hyphae, followed by 6 h antifungal drug treatment, with subsequent XTT assay (Fig. 1A). When exposed to 0.5, 4, and 8 μg mL^−1^ voriconazole, *A. fumigatus* hyphae exhibited quite strong metabolic inhibition (approximately 80%) under iron-replete conditions (minimal medium, MM, containing 18 μM FeSO_4_; Fig. 1B). By contrast, under iron-starved conditions (MM without addition of iron, MM[-Fe]), voriconazole-induced metabolic inhibition was significantly attenuated to approximately 40% (treated with 0.5 μg mL^−1^ voriconazole) or 60% (treated with 4 and 8 μg mL^−1^ voriconazole; Fig. 1B).

To further investigate the relationship between iron concentration and the inhibitory effect of voriconazole against *A. fumigatus* hyphae, the voriconazole-induced metabolic inhibition was assayed under a series of iron concentration gradients. When the FeCl_3_ concentration ranged from 0 to 1 μM, the metabolic inhibitory effect of voriconazole against *A. fumigatus* hyphae increased sharply with rising iron concentration, rising from approximately 40% to approximately 70%. When the FeCl_3_ concentration exceeded 1 μM, the metabolic inhibition of voriconazole against hyphae showed a slow increasing trend reaching approximately 80% at a concentration of 4 μM (MM[-Fe] + 4 µM FeCl_3_), indicating that this iron concentration is sufficient to mediate the metabolic inhibitory effect of voriconazole that is essentially similar to that under MM conditions (Fig. 1C). In comparison, iron starvation exerts a negative effect on the growth of *A. fumigatus*, yet a concentration of FeCl_3_ as low as 0.25 μM is sufficient to sustain the normal growth of *A. fumigatus* hyphae under the tested conditions (Fig. 1C). Collectively, the result demonstrates that the metabolic inhibition rate of voriconazole against *A. fumigatus* hyphae progressively increased along with increasing concentrations of iron.

A similar diminution in metabolic inhibition was seen with itraconazole against *A. fumigatus* hyphae (Fig. S1A). In addition, iron starvation also reduced the metabolic inhibitory effect of amphotericin B against *A. fumigatus* hyphae, though the decrease was minor (less than 10%; Fig. S1B). For caspofungin, its metabolic inhibitory effect against *A. fumigatus* hyphae was reduced only at relatively low concentrations (0.0625 μg mL^−1^ and 0.125 μg mL^−1^; Fig. S1C). These findings collectively demonstrate that iron starvation confers the most significant attenuation of azole activity against *A. fumigatus* hyphae.

### Iron starvation specifically reduces the fungicidal activity of azoles against *A. fumigatus* hyphae

**Fig 2:**
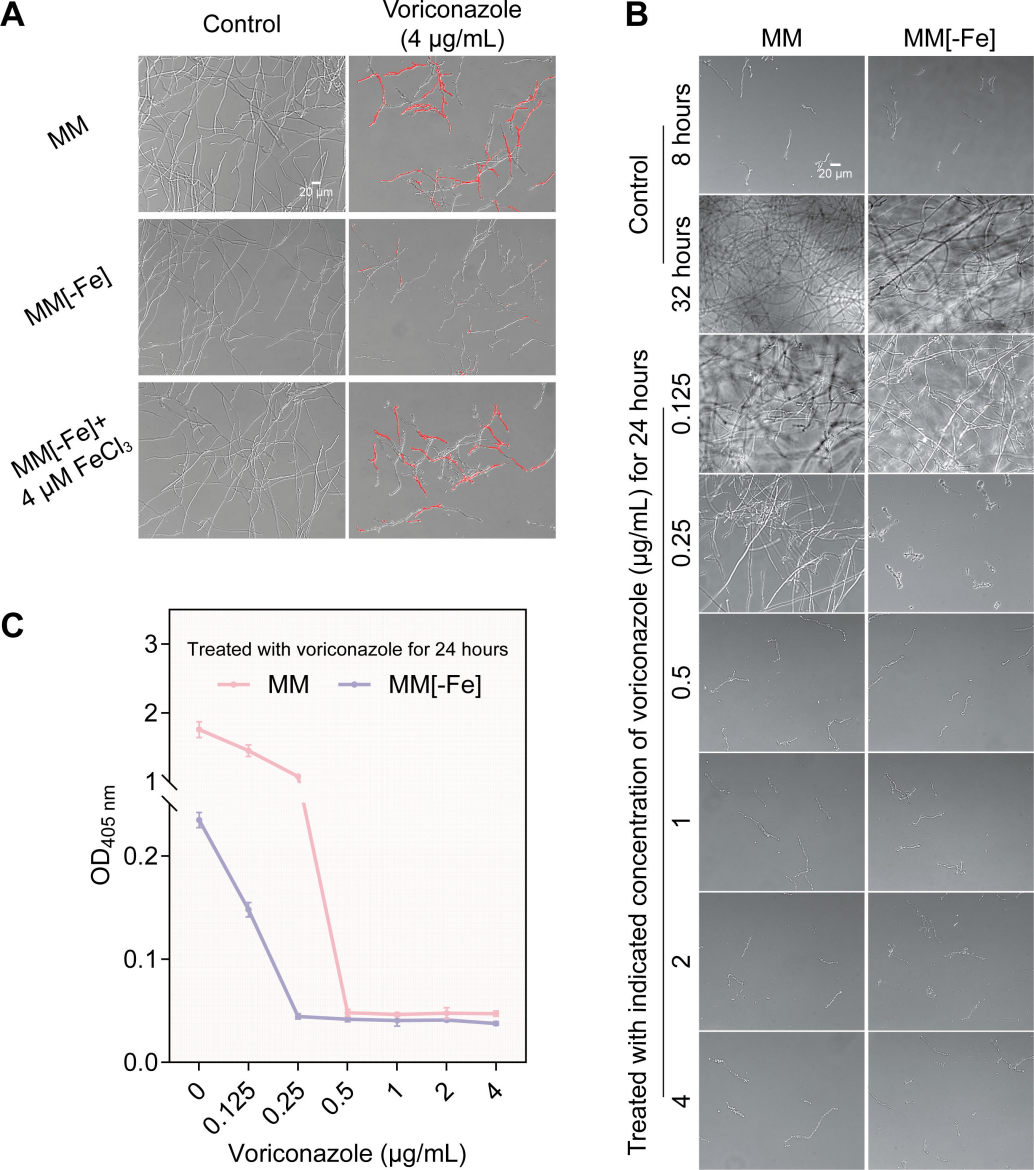
Iron starvation specifically reduces the fungicidal activity of azoles against *A. fumigatus* hyphae. (**A**) Representative images of *A. fumigatus* hyphae stained with the fluorescent dye propidium iodide (PI) under different iron concentrations. Red fluorescence represents the dead hyphae after voriconazole treatment. Conidia of the indicated strains were statically grown at 37°C for 10 h in the indicated medium and then treated with voriconazole at the indicated concentrations at 37°C for an additional 6 h. Finally, hyphae were stained with PI and analyzed with a fluorescent microscope. (**B**) Representative images of voriconazole against *A. fumigatus* hyphal MIC assays under MM and MM[-Fe] conditions. (**C**) The growth of *A. fumigatus* hyphae treated with different concentrations of voriconazole under MM and MM[-Fe] conditions. Conidia of the indicated strains were statically grown at 37°C for 8 h in the indicated medium and then treated with voriconazole at the indicated concentrations at 37°C for an additional 24 h. The growth after treatments was determined by assessing optical density at 405 nm.

Given the above observation, we propose that the reduced metabolic inhibition effect of azoles against *A. fumigatus* hyphae under iron-starved conditions might reflect an enhanced survival capacity at fungicidal azole concentrations. To test this, we first employed fluorescent dye propidium iodide (PI) staining to assess hyphae death following voriconazole treatment under different iron concentrations. Propidium iodide, a membrane-impermeant dye, is excluded by viable cells but enters membrane-compromised cells and exhibits a marked enhancement of red fluorescence. As expected, under MM conditions, the majority of hyphae showed PI-positive after 6 h of 4 μg mL^−1^ voriconazole treatment, whereas the number of PI-positive hyphae under MM[-Fe] conditions was significantly reduced (Fig. 2A). In addition, consistent with the observations from the metabolic inhibition assay, the number of PI-positive hyphae under  MM[-Fe] + 4 µM FeCl_3_ conditions was comparable to that observed under MM conditions (Fig. 2A).

It has been reported previously that the MIC of azole is decreased under iron-starved conditions compared with iron-replete conditions when assayed with conidia (50). We thus investigated whether iron starvation affects the MIC of voriconazole against *A. fumigatus* hyphae. To this end, the conidia of the indicated strains were first statically cultured in the indicated medium for 8 h to form short hyphae, which were subsequently treated with a concentration gradient of voriconazole for an additional 24 h, and the hyphal MIC of voriconazole was ultimately determined by examining microscopic images and optical density assay of the hyphae exposed to each voriconazole concentration. The results show that voriconazole at 0.5 μg mL^−1^ significantly inhibited further hyphal growth of *A. fumigatus* under MM conditions, whereas 0.25 μg mL^−1^ voriconazole was sufficient to achieve a comparable inhibitory effect under MM[-Fe] conditions (Fig. 2B and C). Therefore, iron starvation reduces the azole MIC against both conidia and hyphae in *A. fumigatus*.

Consequently, iron starvation significantly enhanced *A. fumigatus* hyphal survival during azole treatment while not increasing the MIC (but instead decreased) against *A. fumigatus* hyphae. This phenomenon aligns with the established definition of antifungal tolerance.

### Iron starvation specifically enhances the azole tolerance in *A. fumigatus* hyphae

**Fig 3:**
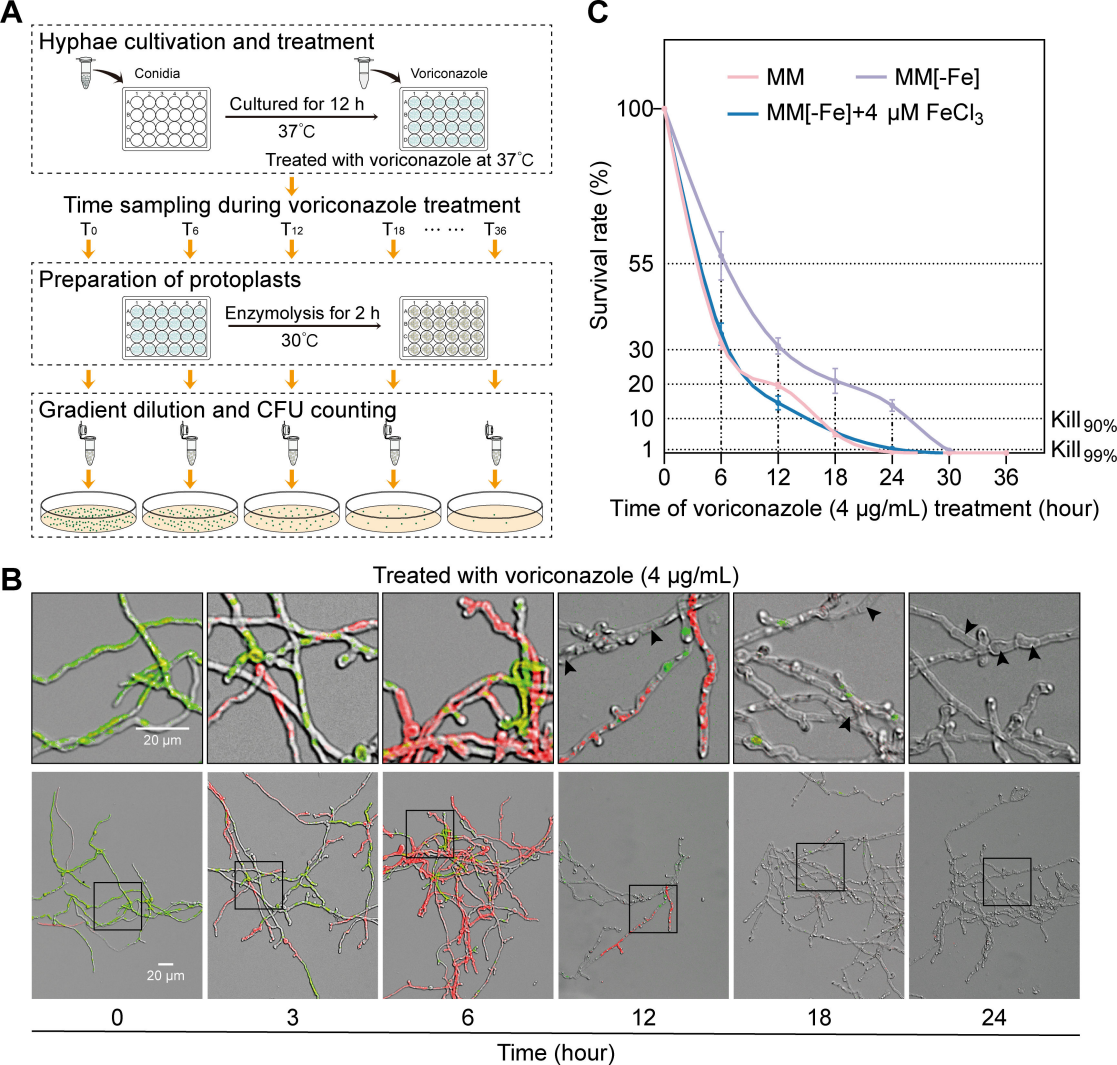
Iron starvation specifically enhances the azole tolerance in *A. fumigatus* hyphae. (**A**) Schematic diagram of protoplast-based hyphal time-kill assay. (**B**) Representative images of *A. fumigatus* hyphae stained with Calcein/PI at indicated time points. The upper panels show magnifications of the framed sections in the lower panels. Green fluorescence represents the surviving hyphae after voriconazole treatment. Red fluorescence represents the dead hyphae after voriconazole treatment. The collapsed hyphae after voriconazole treatment are indicated with black arrowheads. Conidia of the indicated strains were statically grown at 37°C for 12 h in MM and then treated with voriconazole (4 μg mL^−1^) at 37°C for an additional 24 h. The hyphae were stained with Calcein/PI and analyzed with a fluorescent microscope at indicated time points. (**C**) The killing dynamics curves of voriconazole against *A. fumigatus* hyphae under different iron concentrations. Conidia of the indicated strains were statically grown at 37°C for 12 h in the indicated medium and then treated with voriconazole (4 μg mL^−1^) at 37°C for an additional 36 h. At indicated time points after voriconazole treatment, the hyphae were enzymatically digested into protoplasts, and the hyphal survival rate was quantified by CFU counting.

The time-kill assay is a recognized gold-standard method for assessing survival and evaluating antifungal tolerance (19). However, due to the multinucleate nature and physiological heterogeneity of *A. fumigatus* hyphae, effective quantitative methods for assessing hyphal viability have been lacking. We therefore established a protoplast-based method to enable more accurate quantification of hyphal survival rate. In brief, this method involved the enzymatic release of unicellular protoplasts after voriconazole-treated multicellular hyphae, enabling subsequent colony-forming unit (CFU) counting (Fig. 3A). To validate the accuracy of the hyphal time-kill assay using this established protoplast-based method, we concurrently stained the hyphae with live/dead dye Calcein/PI to visualize their viability over time following voriconazole treatment. Calcein is hydrolyzed by intracellular esterases in living cells to produce a fluorescent green signal. Hyphae not treated with voriconazole were almost entirely stained Calcein-positive (Fig. 3B). After 3 h of treatment with 4 μg mL^−1^ voriconazole, the proportions of Calcein-positive and PI-positive hyphae were comparable (Fig. 3B). Within the first 12 h of voriconazole treatment, the number of Calcein-positive hyphal cells gradually decreased as the treatment duration increased, while the number of PI-positive hyphal cells progressively rose (Fig. 3B). After 18 h of voriconazole treatment, only a very small number of hyphal cells remained Calcein-positive, and no PI-positive signals were detected in the field of view (Fig. 3B). Close morphological examination of the hyphae revealed that those treated for 18 h or longer exhibited cellular content leakage and morphological collapse, with the loss of their plump, cylindrical structure (Fig. 3B). Even when such hyphae did not display PI-positive signals, they could be determined as non-viable. When voriconazole treatment was extended to 24 h, both Calcein-positive and PI-positive signals had completely disappeared (Fig. 3B). Correspondingly, in the time-kill assay, the survival rate of hyphae after 3 h of treatment with 4 μg mL^−1^ voriconazole was approximately 55% (Fig. 3C). The time-kill curve showed that the survival rate of hyphal cells also gradually declined with extended voriconazole exposure (Fig. 3C). After 6, 12, and 18 h of voriconazole treatment, the survival rate of hyphal cells decreased to approximately 30%, 20%, and 5%, respectively (Fig. 3C). Finally, the survival rate of hyphal cells after 24 h of voriconazole treatment was nearly 0 (Fig. 3C). The results from the protoplast-based time-kill assay were consistent with the trends shown by the Calcein/PI staining, indicating that this method can accurately reflect the survival of hyphae.

Utilizing this assay, we measured the killing dynamics of voriconazole against *A. fumigatus* hyphae under different iron concentrations. At 4 μg mL^−1^ voriconazole, the time-kill curves for hyphae under iron-replete and iron-starved conditions diverged significantly (Fig. 3C). The minimum duration for killing 99% of the hyphal cells (MDK_99_) was approximately 21 h under MM conditions (Fig. 3C). By contrast, under MM[-Fe] conditions, the MDK_99_ extended to approximately 30 h (Fig. 3C). In addition, the trajectory of the hyphal time-kill curve under MM[-Fe] + 4 µM FeCl_3_ conditions was largely comparable to that under MM conditions, with an MDK_99_ of approximately 24 h (Fig. 3C). Moreover, we assayed the conidial time-kill curves under iron-replete and iron-starved conditions using a previously recommended method (19). To avoid the potential influence of differential endogenous iron content in conidia, which could arise from culture under different iron concentration conditions, conidia harvested from MM and MM[-Fe] were tested separately in the indicated medium. At 4 μg mL^−1^ voriconazole, the trajectories of conidial time-kill curves showed no significant distinction between MM and MM[-Fe] conditions (Fig. S2). The MDK_99_ of the conidia was approximately 18 h in both MM and MM[-Fe] conditions (Fig. S2). Hyphae and conidia therefore exhibit distinct MDK_99_ values, and these data also reveal that hyphal azole tolerance (under both iron-replete and iron-starved conditions) is higher than that of conidia. Collectively, the above results demonstrate that iron starvation can markedly enhance hyphal azole tolerance, whereas this effect is not observed in conidia.

### Iron starvation attenuates the hallmarks of hyphal death induced by azoles

**Fig 4:**
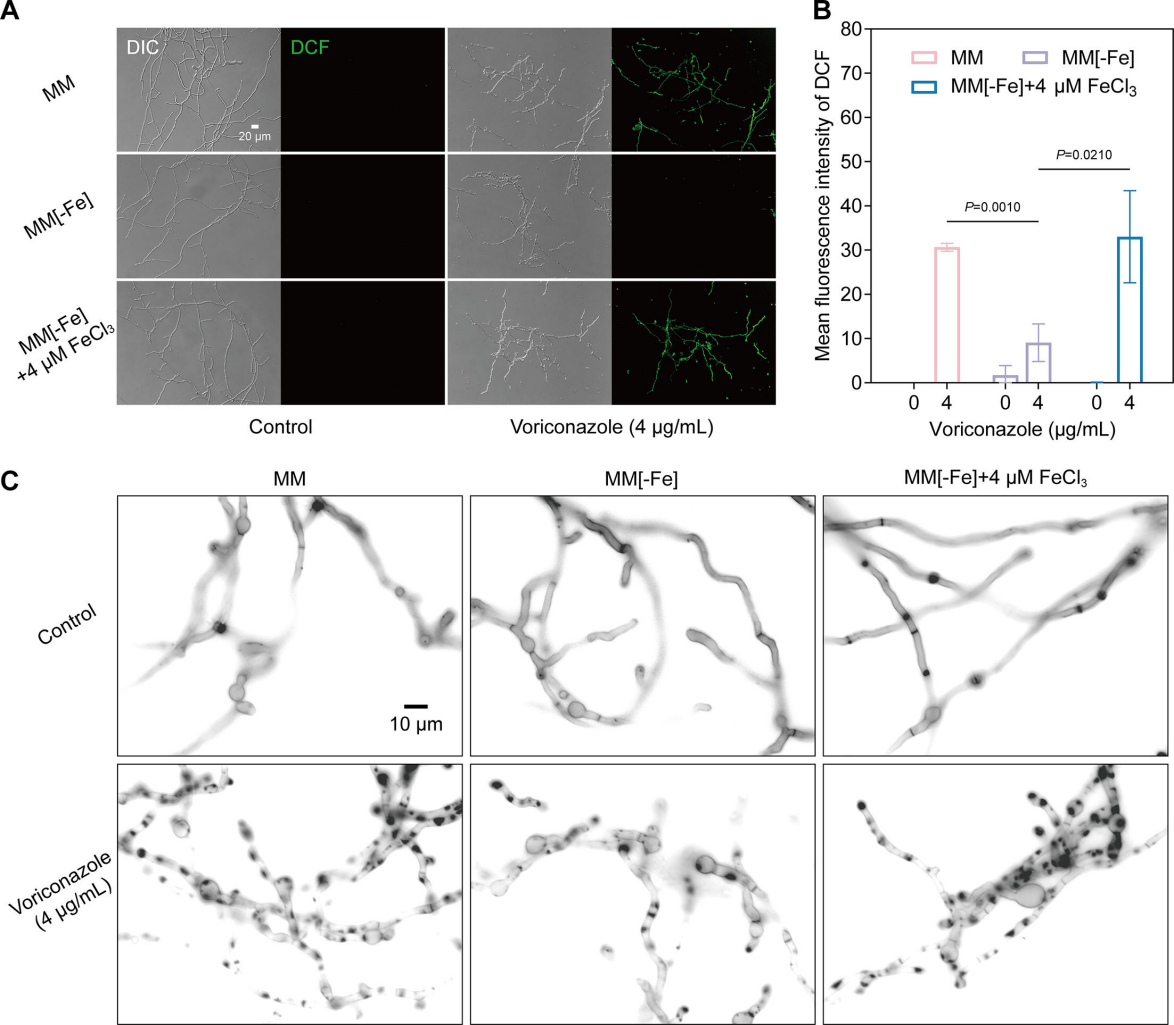
Iron starvation attenuates the hallmarks of hyphal death induced by azoles. (**A**) Representative images of ROS accumulation detected by DCFH-DA staining in *A. fumigatus* hyphae under different iron concentrations. The green fluorescence represents the accumulation of ROS in the hyphae. Conidia of the indicated strains were statically grown at 37°C for 10 h in the indicated medium and then treated with voriconazole at the indicated concentrations at 37°C for an additional 6 h. Finally, hyphae were incubated with DCFH-DA and analyzed with a fluorescent microscope. (**B**) Quantitative analysis of DCF mean fluorescence intensity in panel **A**. Statistical analysis was performed using one-tailed, unpaired *t*-tests. *P* < 0.05 indicates a significant difference, while *P* > 0.05 indicates no significant difference. (**C**) Representative images of staining of carbohydrate patches in *A. fumigatus* hyphae under different iron concentrations. The black spots in the images represent the formed carbohydrate patches. Conidia of the indicated strains were statically grown at 37°C for 10 h in the indicated medium and then treated with voriconazole at the indicated concentrations at 37°C for an additional 6 h. Finally, hyphae were stained with calcofluor white and analyzed with a fluorescent microscope.

The primary mechanism of azoles’ fungicidal effect involves the accumulation of vast ROS and the formation of carbohydrate patches on the cell wall (7, 51). To elucidate the mechanisms by which iron starvation attenuates azole-induced hyphal death, we tested the impact of iron availability on key hallmark events associated with the fungicidal activity of voriconazole. ROS fluorescent probe 2',7'-dichlorodihydrofluorescein diacetate (DCFH-DA) staining showed that under MM conditions, vast ROS accumulation in hyphae occurred after 4 μg mL^−1^ voriconazole treatment for 6 h (Fig. 4A and B). In contrast, this voriconazole-induced ROS accumulation was significantly attenuated under MM[-Fe] conditions (Fig. 4A and B). Under MM conditions, vast carbohydrate patch formation in hyphal cell walls was observed after 4 μg mL^−1^ voriconazole treatment for 6 h (Fig. 4C). Similarly, under MM[-Fe] conditions, the size and number of carbohydrate patches caused by voriconazole treatment were markedly reduced compared to MM conditions (Fig. 4C). Moreover, the hyphal death-associated hallmarks were also observed under MM[-Fe] + 4 µM FeCl_3_ conditions, including ROS accumulation and carbohydrate patch formation (Fig. 4). Collectively, these results reveal that the availability of iron is a central determinant commanding the manifestation of the key characteristics associated with azole-induced hyphal death.

### Decreased mitochondrial function caused by iron starvation confers azole tolerance

**Fig 5:**
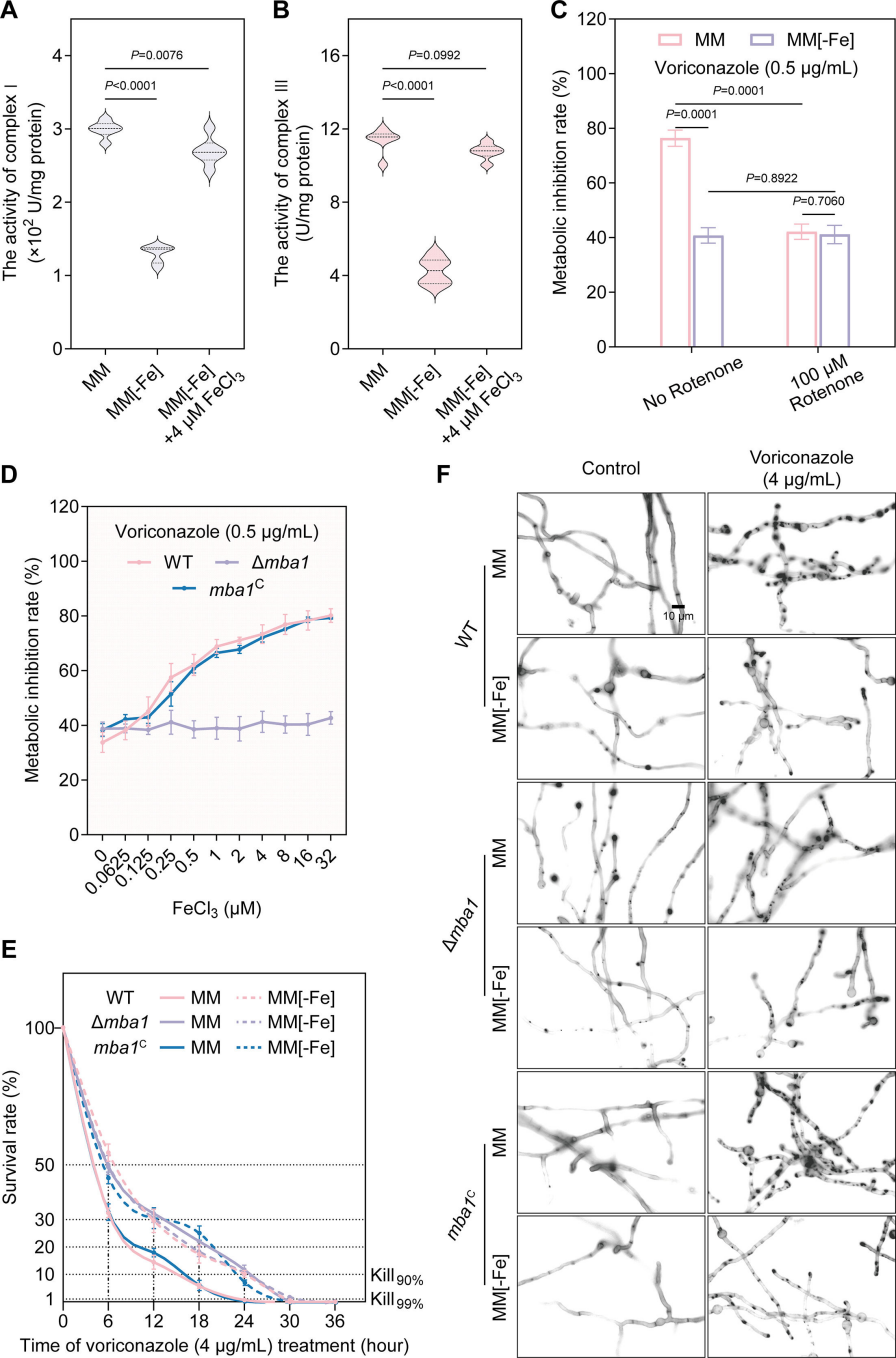
Decreased mitochondrial function caused by iron starvation confers azole tolerance. (**A and B**) The activity assay of *A. fumigatus* mitochondrial ETC complex I (**A**) and complex III (**B**) under different iron concentrations. Conidia of the indicated strains were statically grown at 37°C for 12 h in the indicated medium, followed by hyphae were collected for complex activity assays. Experiments were conducted a minimum of three times, and violin width represents local data density, with horizontal lines denoting medians and interquartile ranges. (**C**) The metabolic inhibition assay of *A. fumigatus* hyphae under synergistic use of complex I inhibitor rotenone and voriconazole. Conidia of the indicated strains were statically grown at 37°C for 12 h in the indicated medium and then hyphae were treated with rotenone in combination with voriconazole at the indicated concentration at 37°C for an additional 6 h. Experiments were conducted a minimum of three times, with each bar indicating the mean ± SD. Statistical analysis was performed using one-tailed, unpaired *t*-tests. *P* < 0.05 represents a significant difference, while *P* > 0.05 represents no significant difference. (**D**) The metabolic inhibition rate of wild-type (WT), Δ*mba1*, and *mba1*^C^ hyphae treated with voriconazole for 6 h under different iron concentrations. Conidia of the indicated strains were statically grown at 37°C for 12 h in the indicated medium and then treated with voriconazole at the indicated concentration at 37°C for an additional 6 h. The metabolic activity was determined by XTT assay, and the metabolic inhibition rate was calculated as the relative metabolic activity of drug-treated cells compared to untreated cells under their respective conditions. (**E**) The killing dynamics curves of voriconazole against WT, Δ*mba1*, and *mba1*^C^ hyphae under MM and MM[-Fe] conditions. Conidia of the indicated strains were statically grown at 37°C for 12 h in the indicated medium and then treated with voriconazole (4 μg mL^−1^) at 37°C for an additional 36 h. At indicated time points after voriconazole treatment, the hyphae were enzymatically digested into protoplasts, and the hyphal survival rate was quantified by CFU counting. (**F**) Representative images of staining of carbohydrate patches in WT, Δ*mba1* and *mba1*^C^ hyphae under MM and MM[-Fe] conditions. The black spots in the images represent the formed carbohydrate patches. Conidia of the indicated strains were statically grown at 37°C for 10 h in the indicated medium and then treated with voriconazole at the indicated concentrations at 37°C for an additional 6 h. Finally, hyphae were stained with calcofluor white and analyzed with a fluorescent microscope.

Iron is crucial for the biogenesis of mitochondrial electron transport chain (ETC) complexes I and III as their assembly relies on iron-sulfur cluster cofactors (42). Iron starvation may impair the assembly and activity of mitochondria ETC complexes I and III, ultimately attenuating mitochondrial function. As expected, iron starvation significantly reduced the activity of both ETC complexes (Fig. 5A and B). Specifically, the activity of complex I was reduced by approximately 60% under MM[-Fe] conditions compared to MM and MM[-Fe] + 4 µM FeCl_3_ conditions (Fig. 5A). Similarly, the activity of complex III showed a comparable reduction of approximately 60% (Fig. 5B). We further speculate that the reduced mitochondrial function might lead to azole tolerance in *A. fumigatus* hyphae. In support of this notion, the addition of rotenone, a specific inhibitor of mitochondria ETC complex I, significantly reduced the metabolic inhibition rate of voriconazole against *A. fumigatus* hyphae under MM conditions, thus recapitulating the iron starvation phenotype (Fig. 5C). In contrast, adding rotenone under MM[-Fe] conditions did not further reduce the metabolic inhibitory effect (Fig. 5C).

To further explore this link, we assayed the metabolic inhibition rate of deletion mutant Δ*mba1* hyphae treated with voriconazole under different iron concentrations. Mba1 is a key mitochondrial assembly factor that is essential for the proper assembly of ETC complex I or complex III and mitochondrial function (8). Notably, the metabolic inhibition rate of voriconazole against Δ*mba1* hyphae remained stable as iron concentrations increased (0–32 μM; Fig. 5D). Even at higher iron concentrations, this strain maintained a relatively low metabolic inhibition rate, a profile consistent with that of the wild-type (WT) hyphae under iron-starved conditions (Fig. 5D). Meanwhile, time-kill assays revealed that under MM conditions, the MDK_99_ of Δ*mba1* hyphae treated with 4 μg mL^−1^ voriconazole was approximately 30 h, nearly identical to that of WT hyphae under MM[-Fe] conditions (Fig. 5E). In contrast, under MM[-Fe] conditions, the time-kill curves of Δ*mba1* hyphae exhibited no significant difference relative to MM conditions, with its MDK_99_ remaining at approximately 30 h and no further extension observed (Fig. 5E). Furthermore, analysis of the hallmarks of azole-induced hyphal death in Δ*mba1* also supports the above observation. Under MM conditions, Δ*mba1* hyphae generated significantly less ROS during azole treatment compared to WT hyphae (Fig. S3). Meanwhile, calcofluor white (CFW) staining revealed that carbohydrate patch formation was also reduced in Δ*mba1* hyphae relative to WT hyphae under MM conditions (Fig. 5F). Importantly, under MM[-Fe] conditions, Δ*mba1* hyphae showed no further decrease in intracellular ROS accumulation and carbohydrate patch formation compared to MM conditions (Fig. S3 and Fig. 5F). These data collectively support that mitochondrial dysfunction induced by iron starvation is a key cause of azole tolerance in *A. fumigatus* hyphae.

Existing studies have shown that deletion of *mba1* confers azole resistance in *A. fumigatus* (8), which might impair the fungicidal effect of voriconazole against hyphae. To rule out this confounding factor, we determined the hyphal MIC of voriconazole against Δ*mba1*. Under MM conditions, the hyphal MIC of voriconazole against Δ*mba1* was 1.0 μg mL^−1^, and this value decreased to 0.5 μg mL^−1^ under MM[-Fe] conditions (Fig. S4). Both concentrations are substantially lower than the 4 μg mL^−1^ fungicidal concentration used in our assays. Thus, it is the azole tolerance, rather than azole resistance, which contributes to the reduced fungicidal efficacy of voriconazole against Δ*mba1* hyphae. Collectively, these results reveal that the decreased mitochondrial function caused by iron starvation confers azole tolerance.

### Coenzyme Q10 can reverse the azole tolerance induced by iron starvation

**Fig 6:**
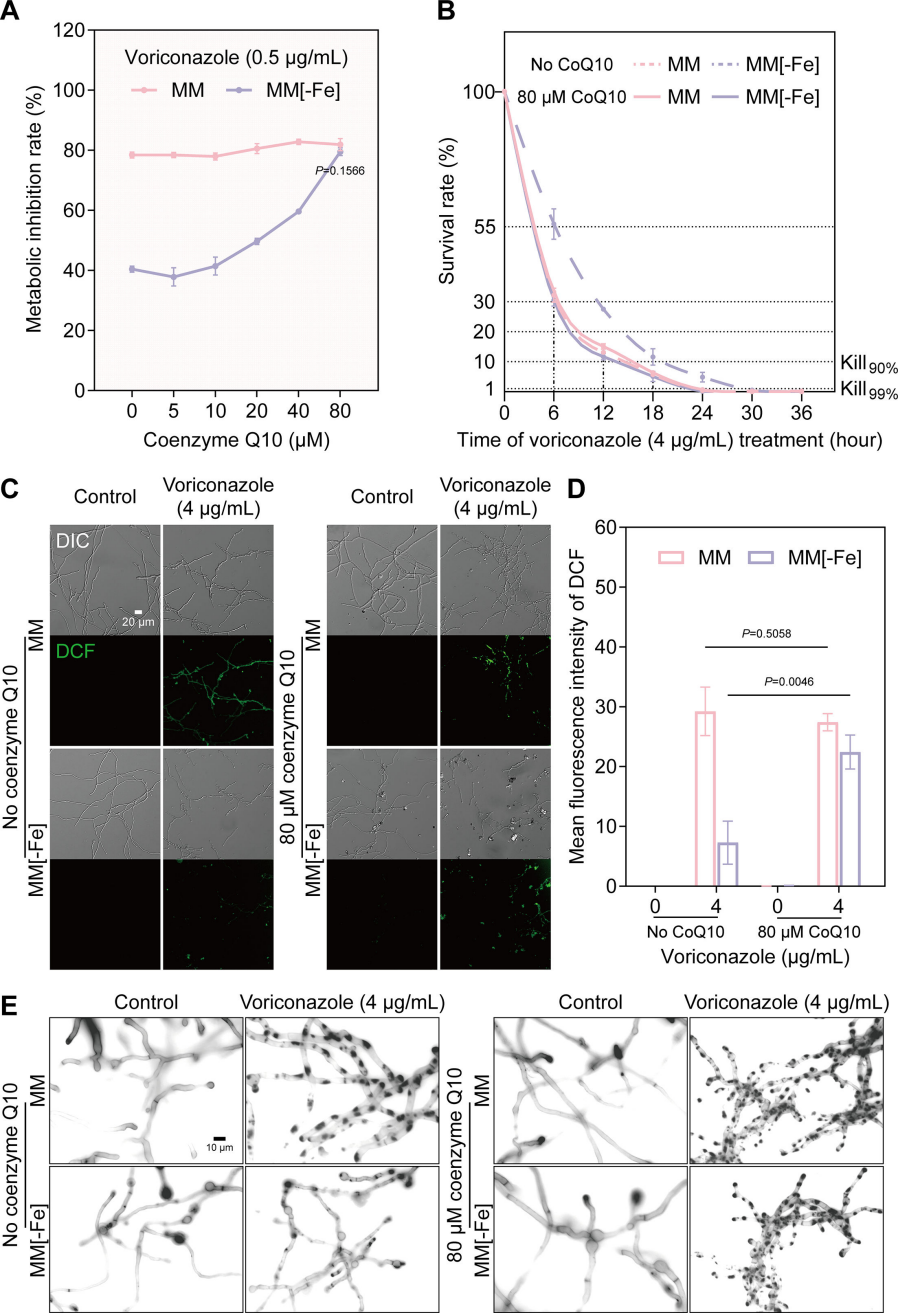
Coenzyme Q10 can reverse the azole tolerance induced by iron starvation. (**A**) The metabolic inhibition assay of *A. fumigatus* hyphae under synergistic use of coenzyme Q10 and voriconazole. Conidia of the indicated strains were statically grown at 37°C for 12 h in the indicated medium and then hyphae were treated with coenzyme Q10 in combination with voriconazole at the indicated concentration at 37°C for an additional 6 h. The metabolic activity was determined by XTT assay, and the metabolic inhibition rate was calculated as the relative metabolic activity of drug-treated cells compared to untreated cells under their respective conditions. Statistical analysis was performed using one-tailed, unpaired *t*-tests. *P* < 0.05 represents a significant difference, while *P* > 0.05 represents no significant difference. (**B**) The killing dynamics curves of coenzyme Q10 in combination with voriconazole against *A. fumigatus* hyphae under MM and MM[-Fe] conditions. Conidia of the indicated strains were statically grown at 37°C for 12 h in the indicated medium and then treated with coenzyme Q10 in combination with voriconazole (4 μg mL^−1^) at 37°C for an additional 36 h. At indicated time points after voriconazole treatment, the hyphae were enzymatically digested into protoplasts, and the hyphal survival rate was quantified by CFU counting. (**C**) Representative images of ROS accumulation detected by DCFH-DA staining in *A. fumigatus* hyphae under synergistic use of coenzyme Q10 and voriconazole. The green fluorescence represents the accumulation of ROS in the hyphae. Conidia of the indicated strains were statically grown at 37°C for 10 h in the indicated medium and then hyphae were treated with coenzyme Q10 in combination with voriconazole at the indicated concentration at 37°C for an additional 6 h. Finally, hyphae were incubated with DCFH-DA and analyzed with a fluorescent microscope. (**D**) Quantitative analysis of DCF mean fluorescence intensity in panel **C**. Statistical analysis was performed using one-tailed, unpaired *t*-tests. *P* < 0.05 represents a significant difference, while *P* > 0.05 represents no significant difference. (**E**) Representative images of staining of carbohydrate patches in *A. fumigatus* hyphae under synergistic use of coenzyme Q10 and voriconazole. The black spots in the images represent the formed carbohydrate patches. Conidia of the indicated strains were statically grown at 37°C for 10 h in the indicated medium and then hyphae were treated with coenzyme Q10 in combination with voriconazole at the indicated concentration at 37°C for an additional 6 h. Finally, hyphae were stained with calcofluor white and analyzed with a fluorescent microscope.

Given that iron starvation induces azole tolerance by impairing the activity of ETC complexes I and III, which may reduce ATP production, we attempted to rescue the potential energy deficit through exogenous ATP supplementation. This treatment, however, failed to restore azole sensitivity (Fig. S5). In fact, when ATP was co-treated with voriconazole, the metabolic inhibition rate of voriconazole against hyphae showed a decreasing trend with increasing ATP concentrations under both MM and MM[-Fe] conditions (Fig. S5). Thus, we speculate that this effect may arise from the specific disruption of mitochondrial iron-dependent enzymatic processes. CoQ10 is a core electron carrier within the inner mitochondrial membrane; it directly participates in and maintains the integrity of the ETC (52). We hypothesized that the addition of CoQ10 could bypass the defects in mitochondrial ETC complexes I and III induced by iron starvation, thereby maintaining the relative stability of mitochondrial electron transport and restoring the susceptibility of *A. fumigatus* hyphae to azoles under iron-starved conditions. As expected, the addition of CoQ10 significantly restored the metabolic inhibitory effect of voriconazole against hyphae under MM[-Fe] conditions (Fig. 6A). In contrast, under MM conditions, CoQ10 supplementation had no effect on the metabolic inhibition rate of voriconazole against *A. fumigatus* hyphae (Fig. 6A). In addition, the addition of CoQ10 showed no significant effect on the MIC of voriconazole against hyphae and conidia under both MM and MM[-Fe] conditions (Fig. S6 and Fig. S7). This suggests that the addition of CoQ10 reduced the tolerance of *A. fumigatus* hyphae to azoles under iron-starved conditions. As expected, the time-kill assay revealed that CoQ10 can reverse azole tolerance induced by iron starvation. Under MM[-Fe] conditions, treatment with 80 μM CoQ10 combined with 4 μg mL^−1^ voriconazole resulted in an MDK_99_ of approximately 22 h for the hyphae, which was nearly identical to the MDK_99_ of them under MM conditions (Fig. 6B). Furthermore, analysis of the hallmarks of azole-induced hyphal death revealed that azole-induced ROS accumulation was significantly higher in CoQ10-treated groups than in controls under MM[-Fe] conditions (Fig. 6C and D). Similarly, CoQ10 also significantly increased the number of azole-induced carbohydrate patches under MM[-Fe] conditions (Fig. 6E). In addition, CoQ10 supplementation can also significantly increase the metabolic inhibition of voriconazole against Δ*mba1* hyphae to the level comparable to that of its complemented strain *mba1*^C^ (Fig. S8A). Time-kill assays further confirmed that CoQ10 co-treatment reduced the MDK_99_ of Δ*mba1* hyphae to a level comparable to that of *mba1*^C^ (Fig. S8B and C).

Collectively, these results demonstrate that iron starvation-induced azole tolerance can be pharmacologically reversed. Targeted CoQ10 supplementation effectively restores azole susceptibility in *A. fumigatus* hyphae under iron-starved conditions, highlighting a potential therapeutic strategy to mitigate iron starvation-driven drug tolerance.

## DISCUSSION

**Fig 7:**
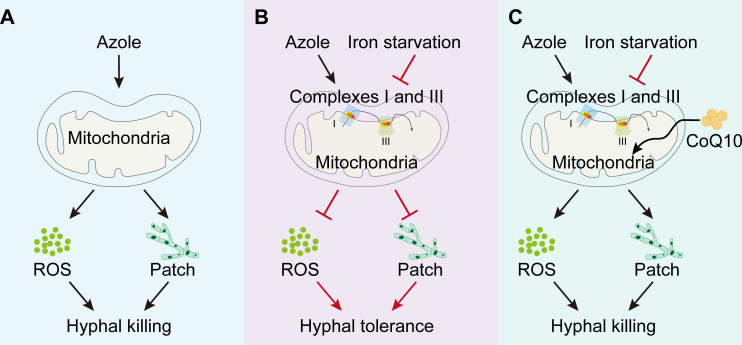
A working model for iron starvation-mediated azole tolerance. (**A**) Azoles kill *A. fumigatus* hyphae primarily by inducing ROS accumulation and carbohydrate patch formation, processes that depend on intact mitochondrial function. (**B**) Iron starvation impairs mitochondrial function by inhibiting complex I and complex III. This reduction in mitochondrial function leads to decreased ROS accumulation and patch formation, which ultimately limits hyphal death and leads to azole tolerance in *A. fumigatus* hyphae. (**C**) This tolerance can be reversed by supplementation with the electron carrier coenzyme Q10.

This study elucidates that iron starvation, a condition that mimics host nutritional immunity, attenuates mitochondrial activity (particularly at complexes I and III). The resulting decrease in mitochondrial function diminishes azole-induced ROS accumulation and carbohydrate patch formation, consequently mitigating irreversible damage to hyphae and ultimately enhancing azole tolerance in *A. fumigatus* hyphae. In addition, we demonstrated that supplementation with the mitochondrial cofactor CoQ10 can effectively restore the fungicidal activity of azoles against hyphae under iron-starved conditions. Based on our observations, we propose a working model delineating how iron starvation confers azole tolerance in *A. fumigatus* hyphae (Fig. 7).

Accurate assessment of antifungal tolerance relies heavily on quantitative fungicidal kinetic parameters such as MDK_99_ (23–26, 53). However, for filamentous fungi, their multicellular and highly interconnected hyphal structures render killing curve methods based on single cells such as conidia inapplicable. Consequently, the field has long relied on conidial testing, which inevitably overlooks the specific responses of the more clinically relevant hyphal form during infection. To accurately reflect the impact of different testing conditions on hyphal azole tolerance, we employed and compared multiple testing methods. The XTT-based metabolic activity assay can quickly and intuitively show the effect of azoles against hyphae, but it cannot distinguish between the fungistatic and fungicidal effects of azoles against hyphae. Calcein/PI staining can reflect the status of hyphae. However, it is difficult to accurately quantify the extent of cell death in hyphal populations. Furthermore, prolonged azole treatment can lead to leakage of intracellular contents (7), resulting in false-negative results. Ultimately, we established and validated a hyphal time-kill assay based on protoplast release. The key advantage of this method is that it enzymatically dissociates the complex multicellular hyphal network into countable single-cell protoplasts. This achieves precise quantification and standardized analysis of the fungicidal kinetics of filamentous fungal hyphal populations.

Strikingly, iron starvation exerts different effects on azole tolerance on distinct developmental stages of *A. fumigatus*. Under iron-starved conditions, the MDK_99_ of voriconazole against conidia shows no significant difference compared to iron-replete conditions. In contrast, under iron-starved conditions, the MDK_99_ of voriconazole against hyphae was significantly prolonged compared to that under iron-replete conditions. This difference indicates that iron starvation specifically reduces the azole tolerance of *A. fumigatus* during the germination process. Iron starvation has previously been shown to transcriptionally reduce mitochondrial activity, including oxidative phosphorylation, via the bZIP transcription factor HapX (54, 55). Consistent with these reports, our findings demonstrate that iron starvation attenuates the activity of mitochondrial ETC complexes I and III, and importantly, the resulting mitochondrial dysfunction plays a critical role in mediating azole tolerance. It has been reported that conidia contain pre-synthesized hydroxyferricrocin that supports their initial germination even under iron-starved conditions, whereas hyphae mainly depend on *de novo* siderophore synthesis and external iron supply (56). In addition, the mitochondrial ETC is largely quiescent in conidia, whereas in hyphae, it is highly active and represents the major source of ATP and ROS (57, 58). Thus, iron starvation may exert a greater impact on mitochondrial function in rapidly growing hyphae than in dormant conidia. Furthermore, iron starvation also exerts different effects on the fungistatic and fungicidal activities of azoles against *A. fumigatus* hyphae. The MIC of azoles against hyphae and conidia was reduced under iron-starved conditions (50), indicating a positive effect of iron starvation on the fungistatic activity of azoles against hyphae and conidia. In contrast, the time-kill assay showed a negative effect of iron starvation on the fungicidal activity of azoles against hyphae. Iron is essential for ergosterol biosynthesis (50, 59), which is consistent with the decrease in azole MICs observed under iron-starved conditions. However, this does not explain the increased azole tolerance of hyphae under iron-starved conditions. Given that mitochondria-derived ROS production and carbohydrate patch formation represent key fungicidal mechanisms of azoles, iron starvation may exert a greater impact on these processes than on ergosterol biosynthesis in hyphae, thereby contributing to azole tolerance in *A. fumigatus* hyphae. Thus, iron plays distinct roles in regulating two biologically linked yet relatively independent processes: growth inhibition and cell death (7). Strikingly, consistent with this regulatory model, relevant studies have demonstrated that complex III dysfunction in *A. fumigatus* resulting from mutations in *bcs1*, *rip1*, and *cycA* leads to a decrease in azole MICs and an increase in azole tolerance (7, 33).

Notably, mutations disrupting mitochondrial complex activity have been linked to azole resistance in *A. fumigatus* (8, 60), which highlights the key role of mitochondrial function in mediating drug responses. Our study clarifies that impaired mitochondrial function is also central to inducing tolerance. Although mitochondria are involved in both resistance and tolerance, the opposing effects of iron starvation on fungistatic and fungicidal activities suggest that the underlying mechanisms are likely distinct. It has been reported that mitochondrial defects can lead to antifungal resistance through multiple mechanisms, such as the activation of calcium signaling pathways via Cox10 deficiency, which upregulates the expression of drug efflux pumps and chitin synthases (60), as well as Cox7c deficiency, which mediates multidrug resistance by increasing heme B accumulation, reducing ROS production, and altering the expression of iron metabolism-related genes (61). Recent studies reveal that complex III plays a role in regulating the metabolic fate of accumulated eburicol, thereby governing the transition from fungistatic to fungicidal activity of azoles (62). Considering iron starvation can also decrease complex III activity and decrease carbohydrate patch formation, we speculate that iron starvation may similarly drive a metabolic shift in toxic sterols, thereby phenocopying the metabolic state induced by complex III mutation. This possibility awaits further targeted metabolomic validation. Mitochondrial ETC complexes I and III are well-established major sources of ROS (63–65). ROS may play a role in both the fungistatic and fungicidal activities of antifungal drugs. However, further research is needed to clarify the specific effects of ROS intensity and duration on these activities.

CoQ10 is a naturally occurring and widely validated safe dietary supplement in humans (66). Its demonstrated ability to reverse iron starvation-induced azole tolerance suggests a highly viable adjunctive therapeutic strategy. In clinical practice, for patients with suspected local iron limitation, combining standard antifungal regimens with CoQ10 may help eliminate hyphal populations in a tolerant state. This could thereby improve cure rates and prevent relapse. Naturally, this concept requires validation in future animal infection models and clinical studies.

## MATERIALS AND METHODS

### Strains, media, and culture conditions

*Aspergillus fumigatus* AF1160 (Δ*ku80* and Δ*pyrG*) was purchased from the Fungal Genetics Stock Center (FGSC); its complemented strain, AF1161 (AF1160::*pyrG*) (67), was used as the parental WT.

The following media were used in this study. Yeast extract glucose (YG) medium (yeast extract, 5 g L^−1^; glucose, 20 g L^−1^; trace elements [ZnSO_4_·7H_2_O, 22 g L^−1^; H_3_BO_3_, 11 g L^−1^; MnCl_2_·4H_2_O, 5 g L^−1^; FeSO_4_·7H_2_O, 5 g L^−1^; CoCl_2_·5H_2_O, 1.6 g L^−1^; CuSO_4_·5H_2_O, 1.6 g L^−1^; (NH_4_)_6_Mo_7_O_24_·4H_2_O, 1.1 g L^−1^; Na_4_EDTA, 50 g L^−1^, adjust pH to 6.5–6.8 with KOH], 1 mL L^−1^) (67). Minimal medium (MM, containing 18 μM FeSO_4_) (nitrate salts [NaNO_3_, 120 g L^−1^; KCl, 10.4 g L^−1^; MgSO_4_·7H_2_O, 10.4 g L^−1^; KH_2_PO_4_, 30.4 g L^−1^], 50 mL L^−1^; glucose, 10 g L^−1^; trace elements, 1 mL L^−1^, adjust pH to 6.5 with NaOH) (67). The MM[-Fe] (MM without addition of iron) used under iron-starved conditions was prepared with iron-depleted trace elements (excluding FeSO_4_·7H_2_O). For MM[-Fe] + 4 µM FeCl_3_ conditions, MM[-Fe] was supplemented with FeCl_3_ in a final concentration of 4 μM.

In general, *A. fumigatus* strains were cultured on YG 2% (wt/vol) agar plates to produce conidia. In conidial time-kill assays, conidia collected from MM and MM[-Fe] 2% (wt/vol) agar plates were tested in parallel. Additionally, the conidia harvested under all conditions were washed with ultrapure water to reduce possible iron residue. All strains were cultured at 37°C in this study.

### XTT assay

Conidia (1 × 10^5^ per mL of medium) were statically cultured in 24-well microplates (500 μL per well) at 37°C for 12 h, and then hyphae were treated with indicated concentrations of voriconazole (S31125, Shanghai Yuanye, Shanghai, China), itraconazole (B25558, Shanghai Yuanye), amphotericin B (V900919, Sigma, St. Louis, USA), or caspofungin (S26841, Shanghai Yuanye) for 6 h. After 6 h of treatment, the medium with drugs was removed, and the hyphae were rinsed once with 500 μL of 1 × PBS. XTT solution (100 μg XTT sodium salt per mL of 1 × PBS with 6.25 μM menadione; XTT sodium salt, S30625, Shanghai Yuanye; menadione, A502486, Shanghai Sangon Biotech, Shanghai, China) was added at 625 μL per well and statically incubated at 37°C for 2 h in a dark chamber. The supernatant of the XTT solution was placed into a 96-well microplate, and the optical density was assessed at 460 nm. To determine the metabolic activity inhibition, drug-treated wells were compared against untreated ones.

### Fungal growth assay under submerged cultural conditions

The growth of *A. fumigatus* under submerged cultural conditions was assessed as previously described (68). In brief, conidia of the indicated strains were mixed with the indicated medium to achieve a total conidial density of 1 × 10^5^ conidia per mL of medium. The mixture was statically cultured in 96-well microplates (180 μL per well) at 37°C, and the hyphae were treated according to the experimental purposes. Finally, the growth was characterized by the optical density at 405 nm.

### Minimal inhibitory concentration (MIC) testing

#### Conidial MIC testing

The conidial MIC of voriconazole was tested using a broth-based microdilution method according to the European Committee for Antimicrobial Susceptibility Testing (EUCAST) E.Def. 9.4 method (69).

#### Hyphal MIC testing

The conidia of the indicated strains were first statically cultured in the indicated medium to generate short hyphae, which were subsequently treated with a series of concentration gradients of voriconazole for an additional 24 h, and the hyphal MIC of voriconazole was ultimately determined by examining microscopic images of the hyphae exposed to each voriconazole concentration. Images were obtained using a Zeiss Axiom Imager A1 microscope (Zeiss, Jena, Germany).

### Determination of the killing dynamics curve

#### The killing dynamics curve for conidia

The killing dynamics curve of conidia is determined through a standardized time-kill assay in liquid medium as previously described (19). Conidia (1 × 10^5^) were exposed to 4 μg mL^−1^ voriconazole for 36 h. At indicated time points (0, 6, 12, 18, 24, 30, and 36 h), samples undergo immediate processing to terminate drug activity: centrifugation followed by supernatant removal, resuspension in voriconazole-free 1 × PBS, and rigorous vortex disperse conidial aggregates. Serial 10-fold dilutions are mixed onto antifungal-free solid medium (2% [wt/vol] agar medium).

#### The killing dynamics curve for hyphae

Conidia (5 × 10^5^) were statically grown for 12 h and then treated with 4 μg mL^−1^ voriconazole for an additional 36 h. At indicated time points (0, 6, 12, 18, 24, 30, and 36 h), samples were immediately subjected to enzymatic digestion at 30°C for 2 h. The enzymatic solution is a per mL of osmotic medium (MgSO_4_, 147.9 g L^−1^; Na_2_HPO_4_, 0.4545 g L^−1^; NaH_2_PO_4_, 0.817 g L^−1^; adjust pH to 5.8 with 1 M Na_2_HPO_4_) containing 3 mg Lysing Enzymes (L3768, Sigma) and 2 mg Yatalase (T017, Takara, Japan). After enzymatic hydrolysis, an equal volume of sorbitol tris-HCl CaCl_2_ buffer (sorbitol, 218.6 g L^−1^; CaCl_2_, 1.1 g L^−1^; tris, 1.2 g L^−1^; adjust pH to 7.5 with HCl) was added, and protoplasts were adjusted to the defined density using hemocytometry. Serial 10-fold dilutions are mixed onto antifungal-free solid medium (0.75% [wt/vol] low-melting-point agar medium containing 1.2 M sorbitol).

After incubation using the above method until visible colony formation (approximately 18 h), CFUs are enumerated. The survival rate was calculated by formulas:


$$Survival\left(\%\right)=\frac{CFUs\;at\;time\;T_{n}}{CFUs\;at\;time\;T_{0}}\times 100\%.$$

Based on the calculated survival rate, locally weighted scatterplot smoothing was used to fit the killing dynamic curves of conidia or hyphae, and the key pharmacodynamic parameters were derived from these curves. Phenotypic classification is based on MDK thresholds. Tolerance is defined by prolonged MDK_99_ relative to a susceptible control.

### Microscopy

#### PI staining, ROS staining, carbohydrate patches staining

Conidia (1 × 10^5^) of the indicated strains were cultured in coverslip with 500 μL indicated medium at 37°C for 10 h, then treated with voriconazole for an additional 6 h. Coverslips were rinsed twice with 1 × PBS followed by subsequent staining procedures for PI, DCFH-DA, and CFW following the same basic protocol: the coverslip was covered with 150 µL of the working dye solution, incubated statically at 37°C in a dark chamber, and then the dye was removed. Specifically, PI (C0080, Shanghai Beyotime, Shanghai, China) was used at 25 μg mL^−1^ for 15 min, DCFH-DA (S0033, Shanghai Beyotime) was used at 10 μM for 45 min, and CFW (S22603, Shanghai Yuanye) was used at 10 μg mL^−1^ for 5 min. Coverslips were then rinsed twice with 1 × PBS. Images were obtained using a Zeiss Axiom Imager A1 microscope. The red, green, and blue fluorescence signals of PI, DCF, and CFW were detected using excitation wavelengths of 532 nm, 488 nm, and 360 nm, respectively.

#### Calcein/PI staining

Conidia (1 × 10^5^) of the indicated strains were cultured in coverslip with 500 μL MM at 37°C for 12 h, then treated with voriconazole (4 μg mL^−1^) for an additional 24 h. At indicated time points (0, 3, 6, 12, 18, and 24 h), samples were first incubated with 150 μL of 0.1% (wt/vol) Tween-20 at 37°C for 30 min to enhance the permeability of the hyphal cell walls to Calcein. Subsequently, 150 μL of the Calcein/PI (C2015S, Shanghai Beyotime) working solution was applied to cover the coverslip, followed by incubation at room temperature for 30 min in a dark chamber. Coverslips were then rinsed twice with 1 × PBS. Images were obtained using a Zeiss Axiom Imager A1 microscope. The green and red fluorescence signals of Calcein and PI were detected using excitation wavelengths of 488 nm and 532 nm, respectively.

### The activity assay of mitochondrial respiratory chain complex I and complex III

Conidia (1 × 10^6^) of the indicated strains were cultured in a dish (Φ90 mm) with 25 mL of the indicated medium at 37°C for 12 h, then hyphae were collected for the mitochondrial respiratory chain complex I and complex III activity assay. Mitochondrial Respiratory Chain complex I/NADH-CoQ Reductase Activity Detection Kit (BC0510, Beijing Solarbio, Beijing, China) and Mitochondrial Respiratory Chain complex III/Cytochrome c Reductase Activity Detection Kit (BC3240, Beijing Solarbio) were used to measure the activity of mitochondrial respiratory chain complex I and complex III, following the manufacturer’s protocols.

## Data Availability

The complete data set is presented in the text and the supplemental material.

## SUPPLEMENTAL MATERIAL

Supplemental Figures mbio.00896-26-s0001.pdf Fig. S1–S8.

ASM does not own the copyrights to Supplemental Material that may be linked to, or accessed through, an article. The authors have granted ASM a non-exclusive, world-wide license to publish the Supplemental Material files. Please contact the corresponding author directly for reuse.
