# Supplementary material for: Iron starvation confers azole tolerance in Aspergillus fumigatus hyphae via mitochondrial function modulation
Source: mBio. 2026 Jun 15;17(7):e00896-26. doi: 10.1128/mbio.00896-26 (PMC13343850; doi:10.1128/mbio.00896-26)
Supplement: Supplemental Figures — Fig. S1–S8. [file mbio.00896-26-s0001.pdf]

# Supplementary Material

## Supplementary Figures

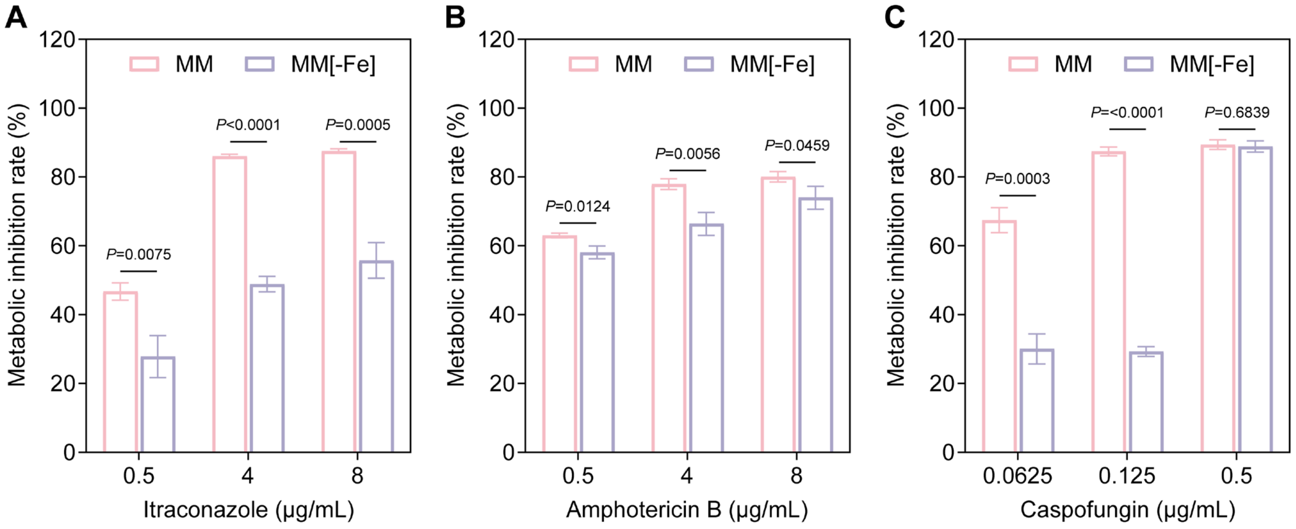


**Figure S1. Iron starvation has a universal effect in reducing the metabolic inhibition of antifungal drugs against *A. fumigatus* hyphae.**

(**A**–**C**) The metabolic inhibition assay of *A. fumigatus* hyphae treated with itraconazole (A), amphotericin B (B) and caspofungin (C) for 6 hours under MM and MM[-Fe] conditions. Conidia of indicated strains were statically grown at 37°C for 12 hours in indicated medium and then treated with antifungal drugs at the indicated concentrations at 37°C for an additional 6 hours. The metabolic activity was determined by XTT assay and the metabolic inhibition rate was calculated as the relative metabolic activity of drug-treated cells compared to untreated cells under their respective conditions. Experiments were conducted a minimum of three times, with each bar indicating the mean ± standard deviation (SD). Statistical analysis was performed using one-tailed, unpaired *t*-tests. $P<0.05$ represents a significant difference, while $P>0.05$ represents no significant difference.

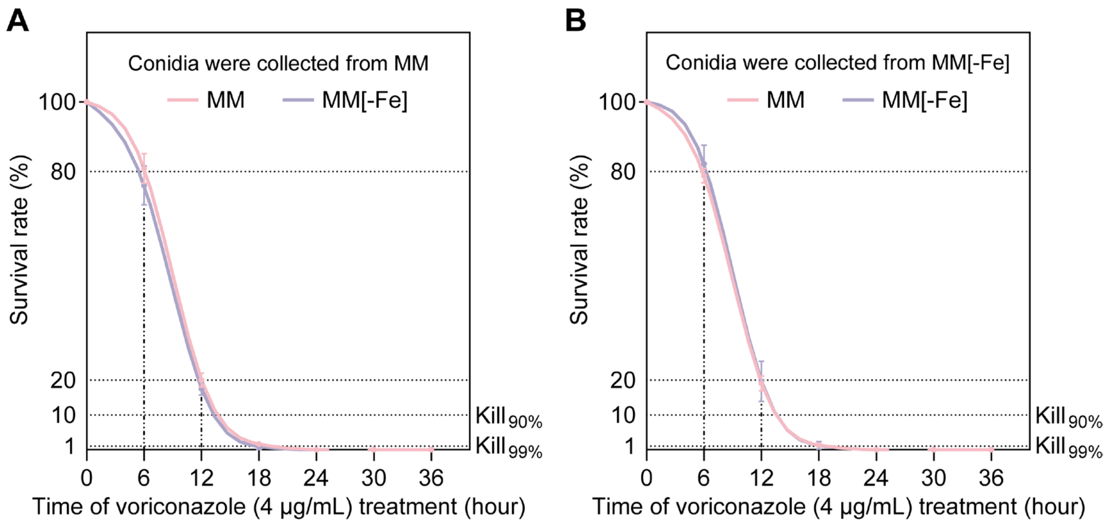


**Figure S2. The killing dynamics curves of voriconazole against *A. fumigatus* conidia under MM and MM[-Fe] conditions.**

Conidia of indicated strains were statically grown at 37°C for 36 hours in indicated medium with voriconazole (4 μg $mL^{-1}$). A, conidia were collected from MM agar plates. B, conidia were collected from MM[-Fe] agar plates. The survival rate of conidia was quantified by CFU counting at indicated time points.

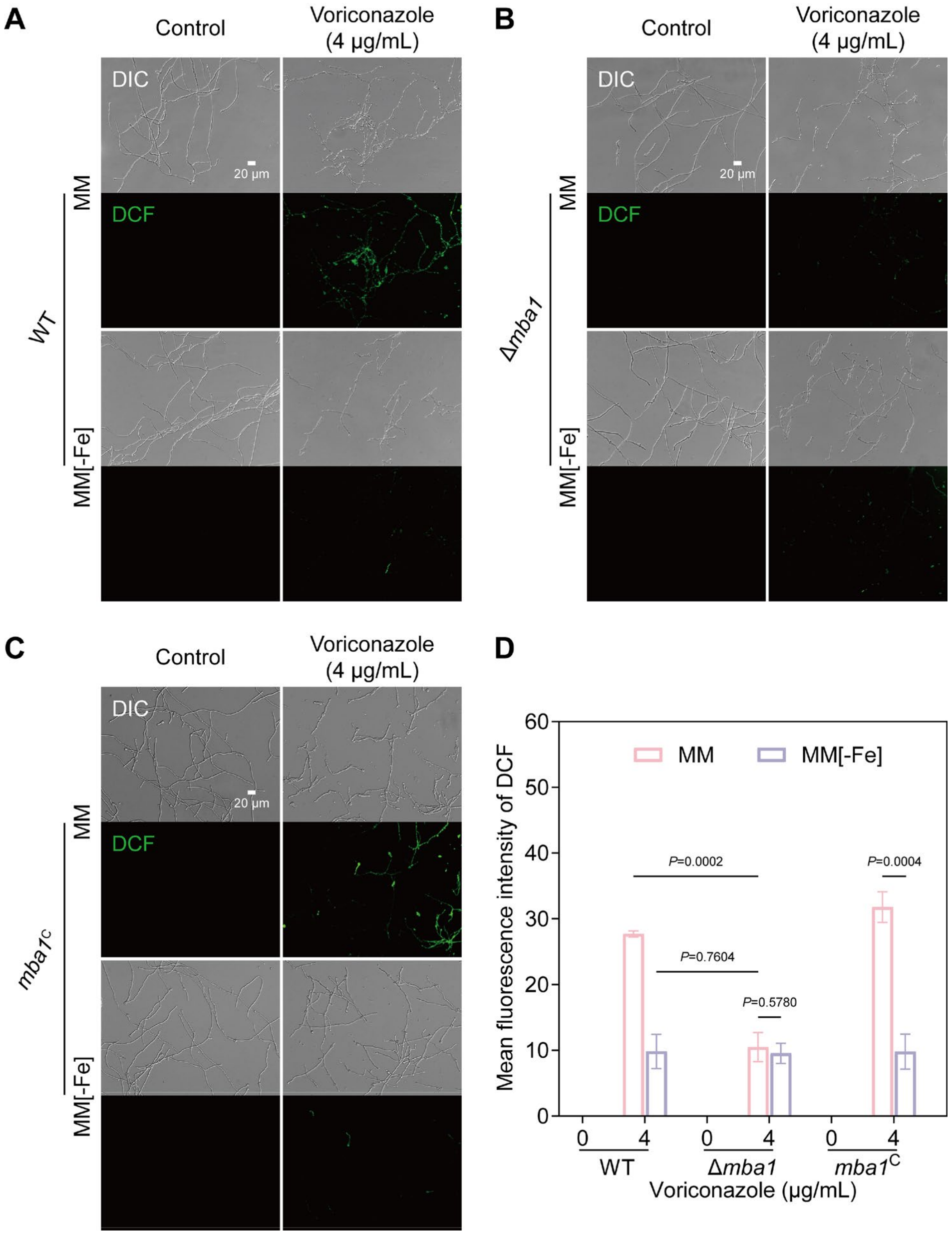


**Figure S3. The assays of ROS levels within WT, Δ*mba1* and *mba1*$^{C}$ hyphae treated with voriconazole for 6 hours under MM and MM[-Fe] conditions.**

(**A**–**C**) Representative images of ROS accumulation detected by DCFH-DA staining in WT (A), Δ*mba1* (B) and *mba1*$^{C}$ (C) hyphae under MM and MM[-Fe] conditions. The green fluorescence represents the accumulation of ROS in the hyphae. Conidia indicated strains were statically grown at 37°C for 10 hours in indicated medium and then treated with voriconazole at the indicated concentrations at 37°C for an additional 6 hours. Finally, hyphae were incubated with DCFH-DA and analyzed with a fluorescent microscope. (**D**) Quantitative analysis of DCF mean fluorescence intensity in (A–C). Statistical analysis was performed using one-tailed, unpaired *t*-tests. $P<0.05$ represents a significant difference, while $P>0.05$ represents no significant difference.

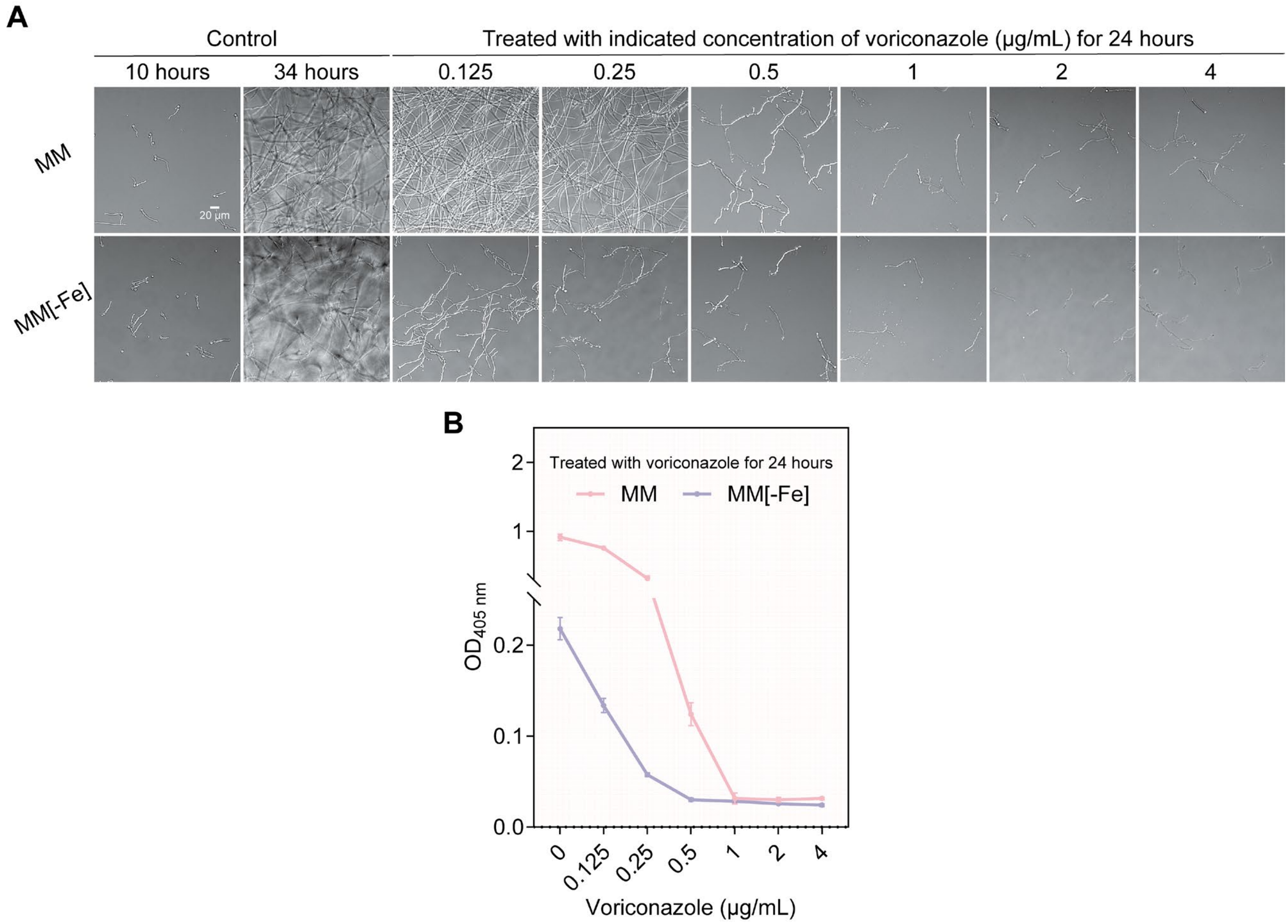


**Figure S4. The MIC assays of voriconazole against Δ*mba1* hyphae under MM and MM[-Fe] conditions.**

(**A**) Representative images of voriconazole against Δ*mba1* hyphal MIC assays under MM and MM[-Fe] conditions. (**B**) The growth of Δ*mba1* hyphae treated with different concentrations of voriconazole under MM and MM[-Fe] conditions. Conidia of Δ*mba1* were statically grown at 37°C for 10 hours in indicated medium and then treated with voriconazole at the indicated concentrations at 37°C for an additional 24 hours. The growth after treatments was determined by assessing optical density at 405 nm.

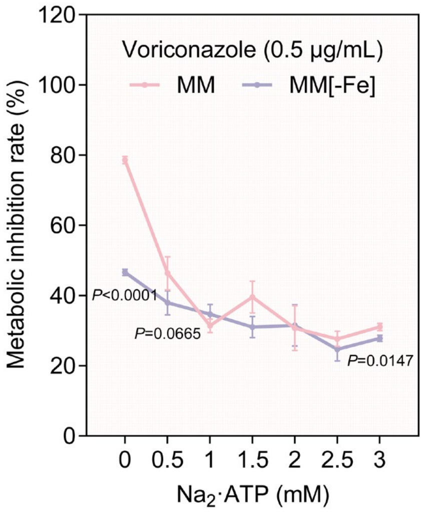


**Figure S5. The metabolic inhibition assay of *A. fumigatus* hyphae under synergistic use of adenosine triphosphate and voriconazole.**

Conidia of indicated strains were statically grown at 37°C for 12 hours in indicated medium and then hyphae were treated with disodium adenosine triphosphate in combination with voriconazole at the indicated concentration at 37°C for an additional 6 hours. The metabolic activity was determined by XTT assay and the metabolic inhibition rate was calculated as the relative metabolic activity of drug-treated cells compared to untreated cells under their respective conditions. Statistical analysis was performed using one-tailed, unpaired *t*-tests. $P<0.05$ represents a significant difference, while $P>0.05$ represents no significant difference.

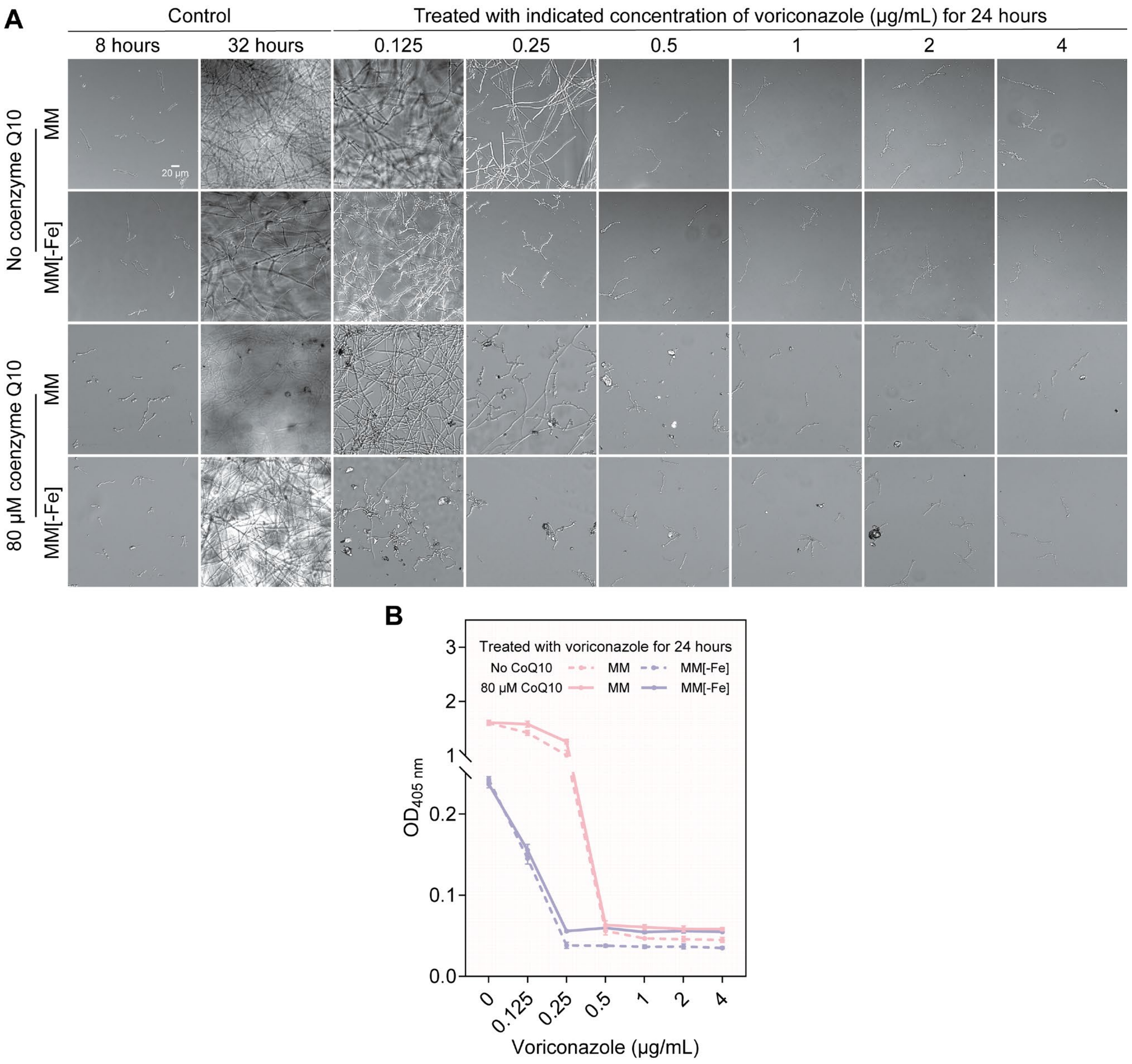


**Figure S6. The MIC assays of voriconazole against *A. fumigatus* hyphae under coenzyme Q10.** (**A**) Representative images of voriconazole against *A. fumigatus* hyphal MIC assays under coenzyme Q10. (**B**) The growth of *A. fumigatus* hyphae treated with different concentrations of voriconazole under coenzyme Q10. Conidia of indicated strains were statically grown at 37°C for 8 hours in indicated medium and then treated with 80 μM coenzyme Q10 in combination with voriconazole at the indicated concentrations at 37°C for an additional 24 hours. The growth after treatments was determined by assessing optical density at 405 nm.

**A** Conidia were collected from MM agar plates

| Iron conditions | MIC of voriconazole against conidia (μg/mL) | |
|---|---|---|
| | No CoQ10 | 80 μM CoQ10 |
| MM | 0.50 | 0.50 |
| MM[-Fe] | 0.25 | 0.25 |

**B** Conidia were collected from MM[-Fe] agar plates

| Iron conditions | MIC of voriconazole against conidia (μg/mL) | |
|---|---|---|
| | No CoQ10 | 80 μM CoQ10 |
| MM | 0.50 | 0.50 |
| MM[-Fe] | 0.25 | 0.25 |

**Figure S7. The MIC assays of voriconazole against *A. fumigatus* conidia under coenzyme Q10.** A, conidia were collected from MM agar plates. B, conidia were collected from MM[-Fe] agar plates.

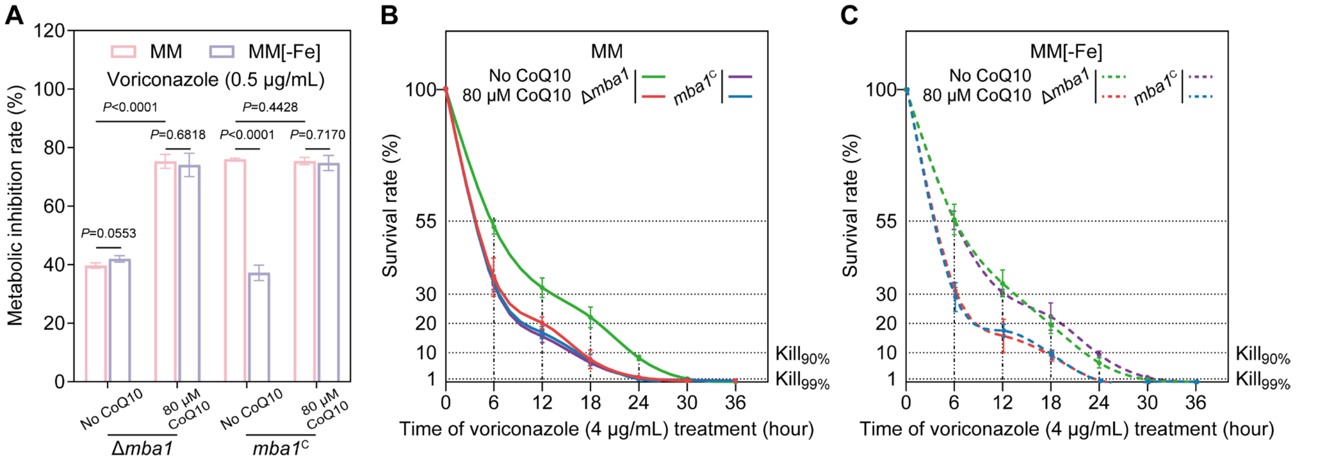


**Figure S8. Coenzyme Q10 can reverse the azole tolerance induced by iron starvation.**

(**A**) The metabolic inhibition assay of Δ*mba1* and *mba1*$^{C}$ hyphae under synergistic use of coenzyme Q10 and voriconazole. Conidia of indicated strains were statically grown at 37°C for 12 hours in indicated medium and then hyphae were treated with coenzyme Q10 in combination with voriconazole at the indicated concentration at 37°C for an additional 6 hours. The metabolic activity was determined by XTT assay and the metabolic inhibition rate was calculated as the relative metabolic activity of drug-treated cells compared to untreated cells under their respective conditions. Experiments were conducted a minimum of three times, with each bar indicating the mean ± SD. Statistical analysis was performed using one-tailed, unpaired *t*-tests. $P<0.05$ represents a significant difference, while $P>0.05$ represents no significant difference. (**B** and **C**) The killing dynamics curves of coenzyme Q10 in combination with voriconazole against Δ*mba1* and *mba1*$^{C}$ hyphae under MM (B) and MM[-Fe] (C) conditions. Conidia of indicated strains were statically grown at 37°C for 12 hours in indicated medium and then treated with coenzyme Q10 in combination with voriconazole (4 μg mL$^{-1}$) at 37°C for an additional 36 hours. At indicated time points after voriconazole treatment, the hyphae were enzymatically digested into protoplasts, and hyphal survival rate was quantified by CFU counting.
